# Architecture of polymorphisms in the human genome reveals functionally important and positively selected variants in immune response and drug transporter genes

**DOI:** 10.1186/s40246-018-0175-1

**Published:** 2018-09-15

**Authors:** Yu Jin, Jingbo Wang, Maulana Bachtiar, Samuel S. Chong, Caroline G. L. Lee

**Affiliations:** 1 NUS Graduate School for Integrative Sciences and Engineering, National University of Singapore, Singapore, 117456 Singapore; 2 Department of Biochemistry, National University of Singapore, Singapore, 119077 Singapore; 3 Division of Medical Sciences, National Cancer Centre, Singapore, 169610 Singapore; 4 Department of Paediatrics, Yong Loo Lin School of Medicine, National University of Singapore, Singapore, 119228 Singapore; 5 Duke-NUS Graduate Medical School, Singapore, 169547 Singapore

**Keywords:** Single-nucleotide variant, Natural selection, Potentially functional SNV, Immune response genes, Drug transporters

## Abstract

**Background:**

Genetic polymorphisms can contribute to phenotypic differences amongst individuals, including disease risk and drug response. Characterization of genetic polymorphisms that modulate gene expression and/or protein function may facilitate the identification of the causal variants. Here, we present the architecture of genetic polymorphisms in the human genome focusing on those predicted to be potentially functional/under natural selection and the pathways that they reside.

**Results:**

In the human genome, polymorphisms that directly affect protein sequences and potentially affect function are the most constrained variants with the lowest single-nucleotide variant (SNV) density, least population differentiation and most significant enrichment of rare alleles. SNVs which potentially alter various regulatory sites, e.g. splicing regulatory elements, are also generally under negative selection.

Interestingly, genes that regulate the expression of transcription/splicing factors and histones are conserved as a higher proportion of these genes is non-polymorphic, contain ultra-conserved elements (UCEs) and/or has no non-synonymous SNVs (nsSNVs)/coding INDELs. On the other hand, major histocompatibility complex (*MHC*) genes are the most polymorphic with SNVs potentially affecting the binding of transcription/splicing factors and microRNAs (miRNA) exhibiting recent positive selection (RPS). The drug transporter genes carry the most number of potentially deleterious nsSNVs and exhibit signatures of RPS and/or population differentiation. These observations suggest that genes that interact with the environment are highly polymorphic and targeted by RPS.

**Conclusions:**

In conclusion, selective constraints are observed in coding regions, master regulator genes, and potentially functional SNVs. In contrast, genes that modulate response to the environment are highly polymorphic and under positive selection.

**Electronic supplementary material:**

The online version of this article (10.1186/s40246-018-0175-1) contains supplementary material, which is available to authorized users.

## Background

Genetic polymorphisms may contribute to the differences in disease risks and drug responses amongst different individuals. Different forms of genetic variants are found in the human genome. Single-nucleotide variants (SNVs) account for more than 90% of genomic variants and are the major form of genetic polymorphisms [1].

Some polymorphisms can affect phenotype. These polymorphisms are likely to alter gene expression or protein function leading to modulation of cellular function and influencing disease risk or drug response. However, to identify the single or a group of causal variants for a particular phenotype from a pool of more than 100 million polymorphisms is like ‘finding a needle in a haystack’ and remains a great challenge since not all genetic variants are functionally important.

While non-synonymous SNVs (nsSNVs) have been extensively investigated as they are the most likely to modulate phenotypes via changing the amino acid composition of proteins, synonymous SNVs (sSNVs) and non-coding variants can also account for phenotypic differences since these variants can affect mRNA stability and transcriptional or translational efficiency and have been associated with gene expression levels in various cell lines and tissues [2–10]. While it may not be feasible to experimentally test every single polymorphism for its function, a variety of bioinformatics tools is now available. These tools can reasonably predict the potential functions of genetic variants, including the likelihood of nsSNVs to disrupt protein structures and/or functions [11–19], SNVs that potentially modify splicing [20, 21] or transcription [22], and SNVs in 3′ untranslated regions (3′UTRs) with potential to alter miRNA target sites [23–25]. There are also comprehensive web tools for predicting various potential functions of both regulatory and coding SNVs, e.g. pfSNP [26] and PupaSNP finder [27]. They can facilitate our understanding of how polymorphisms can lead to phenotype change and help us prioritize the potentially functional SNVs (pfSNVs) for further investigation.

In addition to the above-mentioned predictive bioinformatics tools, signatures of natural selections can also facilitate the identification of causal variants since variants under natural selection are likely to be functionally significant. Patterns of population differentiation were employed to identify 174 candidate gene loci showing signatures of purifying or positive selection [28]. ‘Long-range haplotype’ methods have been employed to identify a list of targets under recent positive selection (RPS) [29]. Another study utilizing HapMap Phase II data found that negative selection preferentially targets non-synonymous sites, while both non-synonymous and 5′ untranslated regions (5′UTRs) show an excess of highly differentiated SNVs, suggesting the evidence of positive selection as well. The authors also reported that variants under selective pressures (either positive or negative) occur more frequently in disease-related genes and are more likely to contribute to disease phenotypes [30].

Although previous reports examined the association of SNVs in regulatory regions with natural selection, these studies were limited. They either merely focussed on only one class of regulatory SNVs (e.g. SNVs within miRBS) [31], on SNVs residing in non-coding regions [32] or within regulatory elements [33] without predicting whether these SNVs alter function (e.g. if a SNV will abolish or create a regulatory site).

In this study, we present the architecture of all genetic polymorphisms of the human genome, focusing on SNVs that are potentially functional and/or positively selected and the pathways that they reside.

## Results

### Polymorphisms are most constrained in coding regions

**Fig. 1:**
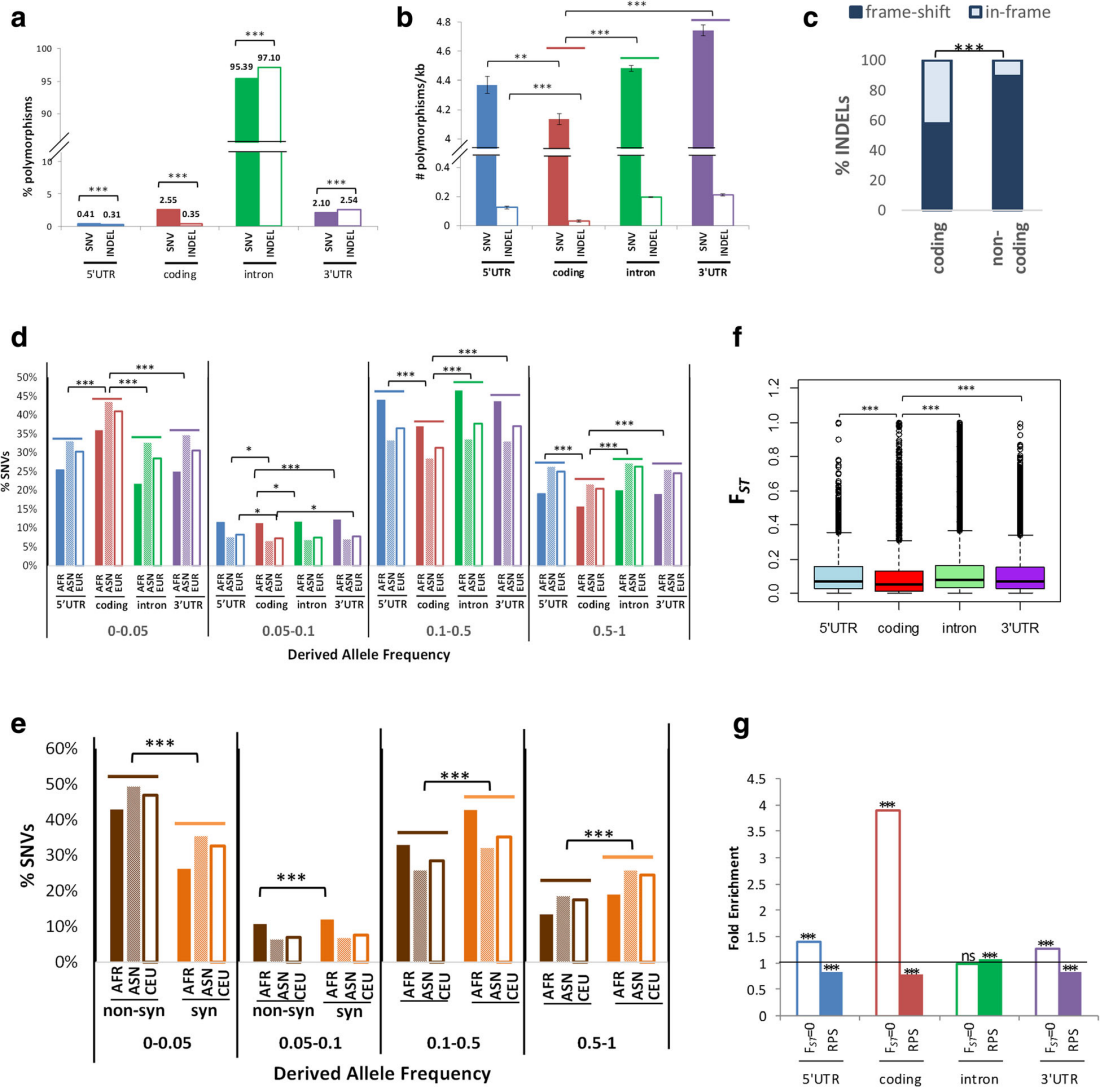
Architecture of polymorphisms in the human genome. **a** Percentage of polymorphisms in different regions (5′UTRs, coding regions, introns, 3′UTRs) of the human genes. **b** Average SNV and INDEL densities (# polymorphisms/kb) in the different regions (5′UTR, coding region, intron and 3′UTR) of a gene in the human genome. Error bars represent the standard errors of the mean SNV and INDEL densities. **c** Percentage of frame-shift and in-frame INDELs in coding and non-coding regions in human genes. Frame-shift INDELS are defined as INDELs whose lengths are not in multiples of three while in-frame INDELs are those whose lengths are in multiples of three. **d** Percentage of SNVs with different DAFs in the four genic regions, as measured in HapMap individuals from African, Asian and European populations. **e** Percentage of synonymous and non-synonymous variants with different DAFs, as measured in HapMap individuals from African, Asian and European population groups. **f** Distribution of *F*_ST_ statistics in four genic regions. SNVs in coding regions show significantly lower median *F*_ST_ compared to the other non-coding regions. **g** Fold enrichment of SNVs showing signatures of negative selection (*F*_ST_ = 0) (open bar) or RPS (shaded bar) in the genic regions. Fold enrichment is determined by the percentage of SNVs with *F*_ST_ = 0 or under RPS in a specific region (e.g. coding region) divided by the percentage of all genotyped SNVs in that region. Coding, coding region; non-syn, non-synonymous; syn, synonymous. AFR, African; ASN, Asian; EUR, European. ***p < 0.001, **p < 0.01, *p < 0.05; ns, not significant

Of the > 14 million polymorphisms in the human genome validated in the dbSNV database (Build 131), 38% of the polymorphisms are within the protein-coding genes while 62% resides in the intergenic regions. More than 95% of the variants within human genes reside within introns (Fig. 1a). Coding polymorphisms constitute ~ 3% of the total polymorphisms within genes, of which 2.55% are SNVs while 0.35% are short insertion/deletions (INDELs) (Fig. 1a). Upon normalization against the length of each genic region, coding regions contain the lowest average densities of both SNVs and INDELs (Fig. 1b). Notably, frame-shift INDELs (i.e. length of INDELs is not in multiples of three) are significantly under-represented in the coding regions compared to non-coding regions in the human genes (*p* value < 0.001 by Fisher’s exact test, Fig. 1c). These data suggest that both SNVs and INDELs are selectively constrained within coding sequences, especially the INDELs with potential to cause frame-shift.

To further investigate the regions within genes that may be most subjected to negative selection pressure, the derived allele frequencies (DAFs) of SNVs in different regions are further compared using allele frequency data of the International HapMap Project individuals. As evident in Fig. 1d, coding regions (red) contain a higher percentage of rare SNVs, defined as having DAF < 0.05 in all the three population groups, namely African, East Asian and European populations. nsSNVs (brown) within the coding region are also enriched with rare alleles compared to sSNVs (orange) (Fig. 1e). As negative selection increases the fraction of rare alleles [34], our results from the analysis of allele frequency data again suggest that coding SNVs, especially nsSNVs, tend to be targeted by negative selection.

Signatures of natural selection are also examined through determining population differentiation using the *F*_ST_ statistics [28] across the different population groups (African, East Asian and European) since high *F*_ST_ is associated with a positive selection [34], while low *F*_ST_ is associated with a negative selection [30]. As shown in Fig. 1f, coding SNVs have lower median *F*_ST_ than SNVs in other regions including 5′UTRs, 3′UTRs and introns (Bonferroni corrected *p* values < 0.001 by Mann-Whitney test). In fact, zero-*F*_ST_ SNVs are significantly over-represented in coding exons (Bonferroni corrected *p* value < 0.001 by Fisher’s exact test) (Fig. 1g, non-shaded bars). Patterns of RPS are examined using linkage disequilibrium (LD) and haplotype-based methods. As shown in Fig. 1g (shaded bars), exonic regions, i.e. 5′UTRs, coding regions and 3′UTRs, are significantly less enriched with RPS SNVs (Bonferroni corrected *p* values < 0.001 by Fisher’s exact test), while introns are more enriched with RPS SNVs (Bonferroni corrected *p* value < 0.001 by Fisher’s exact test).

Taken together, coding regions are generally under strong negative selection pressures as they show the lowest densities of SNVs and INDELs (especially frame-shift INDELs), the highest proportion of rare alleles with less enrichment of RPS SNVs. Notably, coding SNVs are also the least population differentiated.

### Potentially functional SNVs are under natural selections

**Fig. 2:**
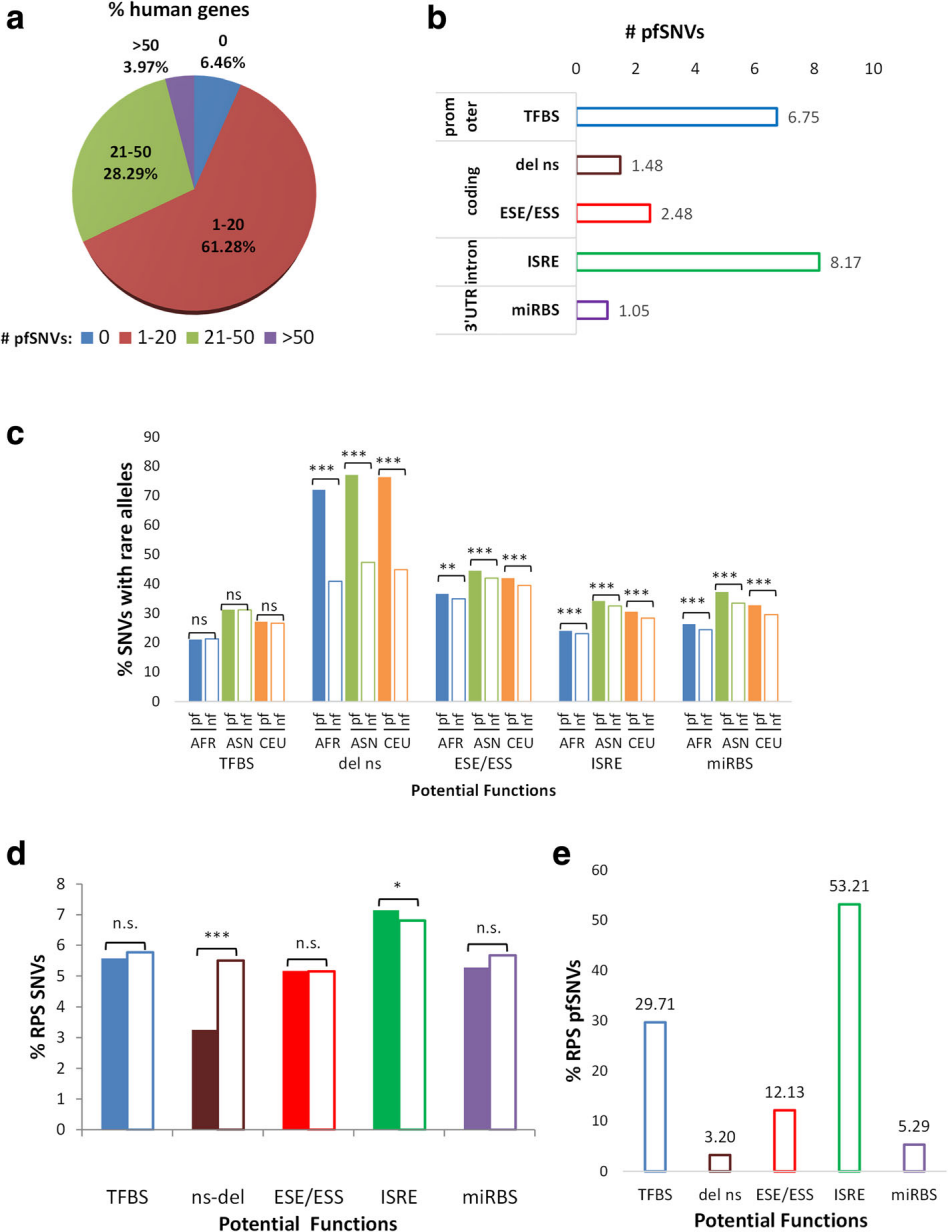
Potentially functional SNVs. **a** Percentage of human genes containing a different number of pfSNVs. **b** Average numbers of pfSNVs with different potential functions in each transcript. **c** Percentage of SNVs with rare alleles (DAF < 0.05) amongst the pfSNVs and nfSNVs in the same genic regions, as determined in HapMap individuals from African (AFR), Asian (ASN) and European (EUR) population groups. **d** Percentage of pfSNVs or nfSNVs under RPS over all pf- or nfSNVs in that specific group. **e** Percentage of SNVs with different functions amongst all the RPS pfSNVs. pf, potentially functional; nf, non-functional; TFBS, SNVs that alter transcription factor binding sites; del ns, potentially deleterious nsSNV; ESE/ESS, SNVs that alter exon splice enhancers/silencers; ISRE, SNVs that alter intronic splicing regulatory elements; miRBS, SNVs that alter miRNA binding sites

The putative functions of SNVs in the various genic and promoter regions are predicted using a variety of bioinformatics algorithms (see Additional file 1: Supplementary Methods). Approximately four hundred thousand (7%) pfSNVs in genic and promoter regions can potentially modulate gene expression and/or function. More than 93% of genes in the human genome contain at least one pfSNV (Fig. 2a). Each gene is predicted to contain an average of seven promoter SNVs capable of altering transcription factor binding sites (TFBS); eight intronic and two coding SNVs that may modulate splicing regulatory elements, i.e. intronic splicing regulatory element (ISRE) and exon splicing enhancers or silencers (ESE/ESS); and one coding SNV that is potentially deleterious to protein function and one SNV in 3′UTR that may alter miRNA binding site(s) (miRBS) (Fig. 2b).

To evaluate if pfSNVs are selectively constrained, the proportions of rare alleles (DAF < 0.05) of pfSNVs and non-functional SNVs (nfSNVs) in a specified region are compared. As evident in Fig. 2c, except for pfSNVs predicted to alter TFBS, most pfSNVs are enriched with rare alleles. Notably, pfSNVs predicted to be deleterious to protein function are more than 1.5-fold more enriched with rare alleles compared to nfSNVs in coding regions indicating that these pfSNVs may be under the strongest negative selection pressure.

Conversely, pfSNVs predicted to be deleterious to protein function are found to be the least significantly enriched with RPS SNVs (Fig. 2d, brown) (Bonferroni corrected *p* value < 0.001 by Fisher’s exact test) consistent with the earlier observation indicating that these pfSNVs are under the strongest negative selection. The other pfSNVs are not under any significant RPS except for pfSNVs predicted to alter ISRE (Bonferroni corrected *p* value = 0.019 by Fisher’s exact test) (Fig. 2d). In addition, more than half of the RPS pfSNVs are predicted to affect ISRE (~ 53%) followed by TFBS (~ 30%) while least RPS pfSNVs (3%) are predicted to be deleterious to protein function (Fig. 2e).

### Highly polymorphic vs conserved genes in the human genome

**Fig. 3:**
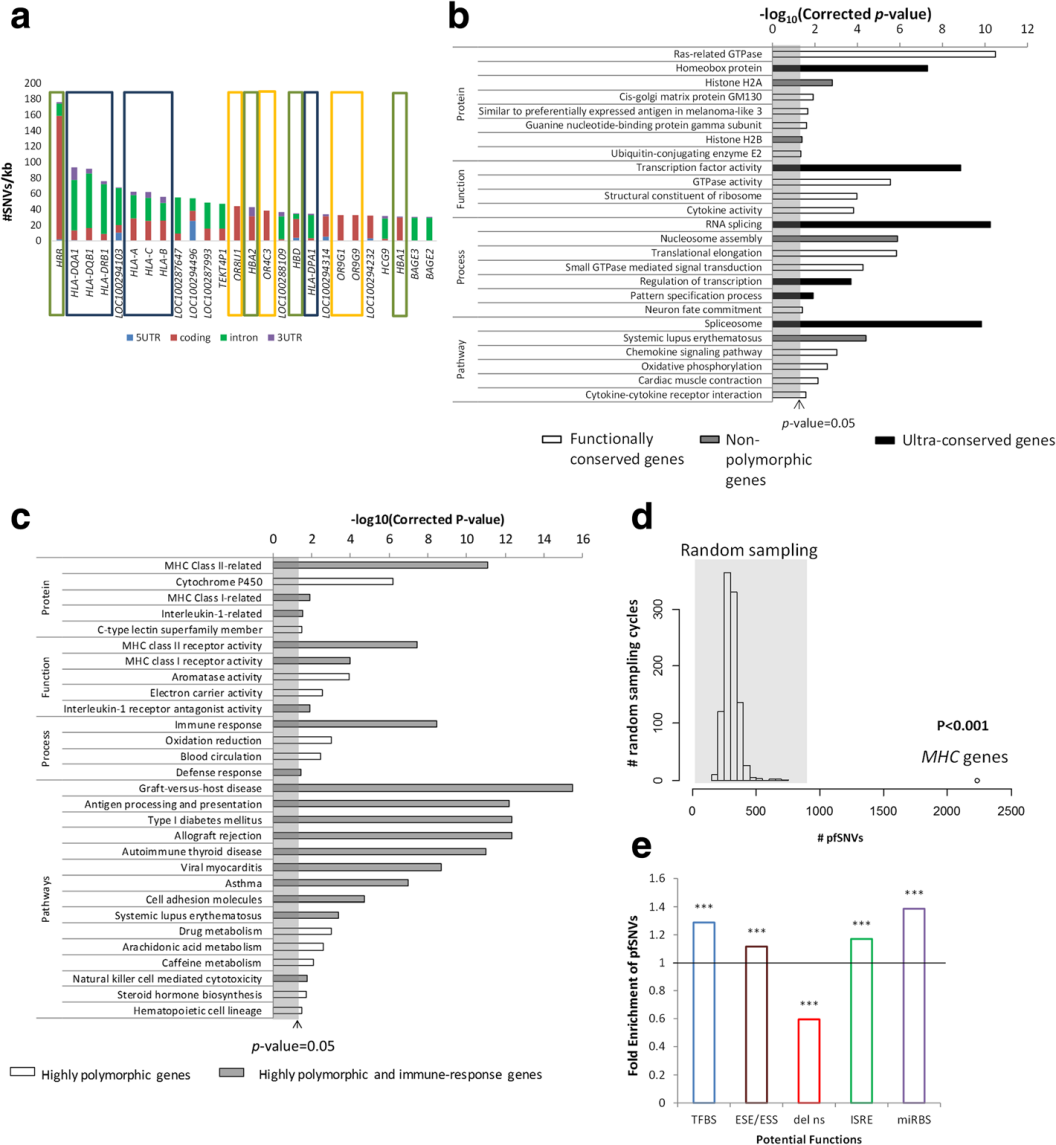
Most polymorphic vs conserved genes in the human genome. **a** Genes having the highest number of SNVs normalized against gene length (> 30 SNVs/kb per gene). Green box: haemoglobin genes; blue box: *MHC* genes; orange box: *OR* genes. **b** Benjamini-corrected *p* values for the significantly enriched functional terms for non-polymorphic genes (grey bars), functionally conserved genes (white bars) which are genes without nsSNVs and coding INDELs as well as ultra-conserved genes (black bars) which are the genes with UCEs within their coding regions. **c** Benjamini-corrected *p* values for the functional terms that are significantly enriched by the highly polymorphic genes. Grey bars: the functional terms related to immune responses. **d** Empirical distribution of the numbers of pfSNVs obtained from 23 genes that are randomly sampled from all the human genes with lengths 3–15 kb for 1000 times. The number of pfSNVs in the 23 *MHC* genes is significantly higher than that in the randomly sampled gene sets (empirical *p* value < 0.001). **e** Enrichment of SNVs with different potential functions in *MHC* class I and class II genes. Fold enrichment is calculated as the percentage of pfSNVs in specific genic regions (e.g. coding SNVs that may alter ESE/ESS) in the *MHC* class I and class II genes against that for all the human genes. Deviation from one indicates that the pfSNVs are over- or under-represented in the *MHC* genes

Amongst > 20,000 genes in the human genome, beta haemoglobin (*HBB*) gene is the most polymorphic gene, containing approximately 176 SNVs per kilobase (kb) with the highest density of SNVs within its coding region (Fig. 3a, red) (570 SNVs/kb). Several other haemoglobin genes (in green boxes) are also amongst the most polymorphic genes in the human genome with the majority of their SNVs residing within coding exons (red). Other highly polymorphic genes include the *MHC* family of genes (blue box) with most of their SNVs residing within introns (Fig. 3a, green) as well as the olfactory receptor (*OR*) gene family (orange box) where all the SNVs are also found within the coding region (Fig 3a, red).

The density of SNVs within each gene, normalized against their length, is determined for all > 20,000 protein-coding genes in the human genome. Most genes have approximately four SNVs per kilobase. Although ~ 97% of genes carry at least one SNV, 149 genes do not contain any polymorphism as SNV or INDEL. More than half of these 149 non-polymorphic genes were yet to be annotated. Nonetheless, the annotated non-polymorphic genes are significantly over-represented in histone 2A and 2B families and involved in nucleosome assembly (Fig. 3b, grey shaded; Additional file 1: Table S1).

We then focus on polymorphisms within the coding region since this region encodes the functional protein. Nearly 20% (4389) of genes in the human genome are found to be functionally conserved with no nsSNVs nor coding INDELs. These genes are enriched in various categories including GTPases and translational elongation (Fig. 3b, unshaded; Additional file 1: Table S2). Seventy genes are found to carry ultra-conserved elements (UCEs) [35] in their coding regions; hence, these genes are evolutionarily conserved. These ultra-conserved genes include the homeobox proteins and are primarily involved in the transcription factor activity, RNA splicing and pattern specification (Fig. 3b, shaded black; Additional file 1: Table S3).

Taken together, genes involved in the basic fundamental biological process, for example, gene regulation, are highly conserved during evolution, being least polymorphic within the human species as well as between species.

### Highly polymorphic genes are mainly involved in immune responses

A total of 512 highly polymorphic genes (see Additional file 1: Supplementary Methods) are identified. These genes are the most significantly over-represented in immune response pathways, as well as in the pathogenesis of a number of autoimmune diseases, including Graft-versus-host disease and type I diabetes mellitus (Fig. 3c, shaded grey; Additional file 1: Table S4). They are primarily in the *MHC* class I-related and class II-related protein families, involved in antigen presentation and processing. Majority of the *MHC* class I and class II genes are located on chromosome 6q21.3 which is the most polymorphic region in the human genome and facilitate the generation of diverse antigens to confer a selective advantage to fight infection [36]. The number of pfSNVs in the *MHC* genes (2234) is higher than the average number of pfSNVs in the other human genes (306). To determine if pfSNVs are significantly over-represented in this *MHC* gene family of 23 genes, sampling of 23 random human genes with similar gene length is performed 1000 times. The number of pfSNVs in the 23 random genes for each cycle is plotted to obtain an empirical distribution. The *MHC* family of genes, with 2234 pfSNVs, is found to carry significantly more pfSNVs than 1000 different sampling of 23 random human genes (empirical *p* value < 0.001 by random sampling test) (Fig. 3d). Interestingly, despite the enrichment of pfSNVs in the *MHC* gene family, there are significantly fewer nsSNVs predicted to be deleterious in the *MHC* family of genes, compared to other human genes (Bonferroni corrected *p* value < 0.001 by Fisher’s exact test) (Fig. 3e). Hence, this family of proteins can nimbly respond to different infection, through a diversity of different regulatory mechanism, including differential transcription factor/miRNA binding/splicing.

Lastly, highly polymorphic genes are also significantly enriched in drug metabolism, cytochrome P450 (*CYP450*), arachidonic acid and caffeine metabolism pathways (Fig. 3c, Additional file 1: Table S4).

### Drug response genes are most affected by potentially deleterious polymorphisms in coding regions

**Fig. 4:**
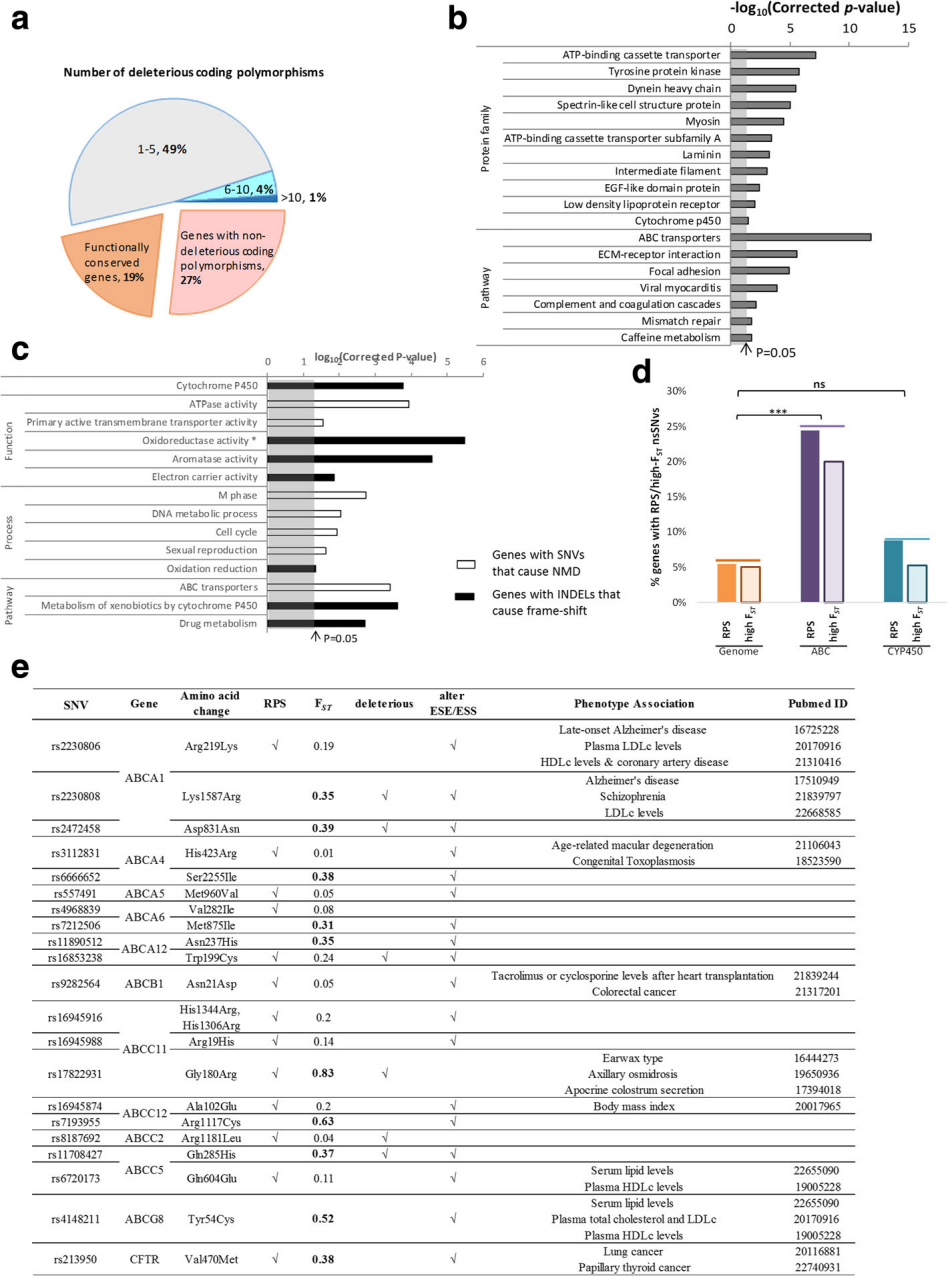
Distribution of potentially deleterious coding polymorphisms in human genes. **a** Percentage of genes with different numbers of potentially deleterious coding polymorphisms in their coding regions. Genes without any potentially deleterious coding polymorphisms are divided into two groups: (1) functionally conserved genes, i.e. genes with no nsSNV nor INDELs in coding regions; (2) genes carrying non-deleterious SNVs in their coding regions. **b** Benjamini-corrected *p* values for the functional terms that show enrichment of the genes with more than five potentially deleterious nsSNVs. **c** Benjamini-corrected *p* values for the functional terms that show enrichment of the genes with SNVs that cause NMD (non-shaded) and the genes with coding INDELs that cause frame-shift (black). **d** Percentage of genes with RPS nsSNVs and genes carrying nsSNVs with high *F*_ST_ (> 0.3) in the whole genome, *ABC* transporter and *CYP450* family. **e** Recently positively selected and/or population-differentiated nsSNVs in the *ABC* transporters. *F*_ST_ scores in bold indicate *F*_ST_ > 0.3. *Oxidoreductase activity: oxidoreductase activity, acting on paired donors, with incorporation or reduction of molecular oxygen, reduced flavin or flavoprotein as one donor, and incorporation of one atom of oxygen

Although > 90% (20,890/22,333) of the genes in the human genome have pfSNVs, ~ 54% contain at least one potentially deleterious coding polymorphism (Fig. 4a, shaded blue, dark blue and grey) while ~ 19% are functionally conserved with no nsSNVs nor coding INDELS (Fig. 4a, shaded orange). Potentially deleterious coding polymorphisms are under the strongest negative selection as suggested earlier (Fig. 2c) since they can potentially have a drastic effect on protein function. Approximately 5% (1104/22,333) of all genes in the human genome are highly enriched with more than five potentially deleterious nsSNVs in their coding regions (Fig. 4a, shaded blue and dark blue). These genes are significantly enriched in the ATP-binding cassette (*ABC*) transporter and the *CYP450* families, which play important roles in drug transport and metabolism (Fig. 4b, Additional file 1: Table S5). Notably, most of the common drug metabolizers including *CYP3A4* [37], *CYP1A1* [38] and *CYP2D6* [39] contain more than five potentially deleterious nsSNVs, while the *CYP* genes that metabolize endogenous substance, e.g. *CYP51A1* [40], are not affected by any potentially deleterious nsSNVs. Similarly, important xenobiotic transporters including *ABCB1*, *ABCC1* and *ABCG2* [41] have 8, 11 and 7 potentially deleterious nsSNVs, respectively, while genes in *ABCD* subfamily, which are peroxisomal transporters for very long chain fatty acids [42], contain fewer (1–3) potentially deleterious nsSNVs. In addition to drug metabolizer and transporter, other protein families enriched in genes with more than five predicted deleterious nsSNVs include tyrosine protein kinases, dynein heavy chains, spectrins and myosins.

Notably, not only are the *ABC* transporters significantly enriched in predicted deleterious coding SNVs (Fig. 4b), they are also enriched with nsSNVs that are predicted to cause nonsense-mediated decay (NMD) resulting in the degradation of the mRNA transcripts with premature stop codon (Fig. 4c, clear bars; Additional file 1: Table S6). Genes containing nsSNVs predicted to cause NMD are also significantly enriched in cell cycle processes including mitosis (Fig. 4c, clear bars; Additional file 1: Table S6).

While the *ABC* transporters are significantly enriched with nsSNVs predicted to cause NMD, the other family of genes involved in drug response, the *CYP450*, is significantly enriched with genes having another form of deleterious polymorphism, namely, INDELs that cause frame-shift, which have deleterious effect on protein function (Fig. 4c, black bars; Additional file 1: Table S7). Taken together, genes involved in the xenobiotic response, including drug transport and metabolism, are significantly enriched with potentially deleterious coding polymorphisms.

Signatures of natural selection on the nsSNVs in drug-response genes are investigated. Interestingly, unlike the *CYP450* family (5/57 genes), not only are the *ABC* transporters enriched with potentially deleterious coding polymorphisms, they are also significantly enriched (*p* value < 0.001 by Fisher’s exact test) with genes carrying nsSNVs under RPS (11/45 genes) (*p* value = 0.24 by Fisher’s exact test) compared to the other genes in the human genome (Fig. 4d). As genes under positive selection also show significant population differentiation [34], we evaluate if the drug response genes are also enriched with nsSNVs that show significant population differentiation (*F*_ST_ > 0.3). Similar to the above observations, Fisher’s exact test revealed that the *ABC* transporters (9/45) (*p* value < 0.001 by Fisher’s exact test) but not the *CYP450* genes (3/57) (*p* value = 0.76 by Fisher’s exact test) are significantly enriched with nsSNVs that show significant population differentiation (Fig. 4d). Hence, the nsSNVs in the *ABC* transporter family are under strong positive selection pressure.

As evident from the table in Fig. 4e, all, except one (rs4968839), of the nsSNVs at the *ABC* transporter family, which showed evidence of RPS or significant population differentiation, are predicted to either have a potentially deleterious effect on protein function or alter ESE/ESS modulating the proportion of the different splice forms. Notably, > 40% of these nsSNVs have been reported to be significantly associated with various phenotypes including clinically relevant ones [43–59] (Fig. 4e), highlighting the functional importance of the nsSNVs under natural selection at the *ABC* transporter gene family.

## Discussion

In this study, we comprehensively investigate the architecture of genetic polymorphisms in the human genome and demonstrate that polymorphisms in coding regions, especially those affecting protein sequences and/or functions, are the most constrained in the human genome, consistent with previous observations [30]. In particular, frame-shift INDELs in coding regions are under strong purifying selection, consistent with the previous observation of the strongest depletion of frame-shift INDELs in coding regions, which are enriched with gene expression association possibly contributed by NMD [60].

Through the interrogation of nine global populations, we demonstrate that the median *F*_ST_ of SNVs at the coding regions is lower than that of the other regions of the genome (Fig. 1f). Moreover, the observations that coding regions have the lowest SNV density (Fig. 1b), excess of rare alleles (Fig. 1d) and enrichment of SNVs with no population differentiation (Fig. 1g) all indicate that coding SNVs are constrained by purifying selections. This is further strengthened by the observation that potentially deleterious nsSNVs show enrichment of rare alleles, compared to non-deleterious nsSNVs (Fig. 2c). Furthermore, coding regions contain significantly fewer INDELs that cause frame-shift (Fig. 1c). Hence, polymorphisms predicted to be deleterious to protein functions are under the strongest purifying selection.

In addition to the potentially deleterious nsSNVs, the potential functions and signatures of natural selections in the other polymorphisms are also investigated. Through computational prediction of the potential functions of SNVs, we observe that significantly more SNVs are predicted to alter TFBS than to code for a potentially deleterious nsSNV (Fig. 2b). Additionally, except for SNVs affecting TFBS, the other pfSNVs show more significant enrichment of rare alleles than nfSNVs in the same regions (Fig. 2c). Hence, pfSNVs are more constrained than the other SNVs in the same region, perhaps because they affect the functionally important regulatory sites. This observation is congruent with previous studies that reported stronger negative selection on conserved miRBS than other conserved 3′UTR sequences [31], though different prediction algorithms and SNV data were used. Levenstien and Klein also reported similar observation that SNVs in a few functional classes, e.g. non-synonymous, methylation sites and miRBS, are under negative selection compared to genome, and suggested that they are promising candidates for functional characterization [61]. While these previous studies examined SNVs residing within regulatory consensus sites of the promoter, this study focuses on SNVs that are predicted to either disrupt or create regulatory sites. Hence, the negative selective pressure on several different classes of pfSNVs suggests that pfSNVs are likely to influence gene functions and contribute to phenotypic changes.

This study also highlights that ‘master regulators’ of gene expression tend to be functionally conserved and maintained during evolution, while regulation of specific target genes is less constrained and flexible. This is evident from the observation that ‘master regulator’ involved in general gene regulation including epigenetics (e.g. histones), transcription/translation and splicing is significantly enriched with non-polymorphic, functionally conserved and ultra-conserved genes (Fig. 3b). In contrast, an average gene contains more SNVs affecting its own regulation than altering its function as evident from the observed enrichment of SNVs predicted to alter TFBS at promoters, ESE/ESS in coding regions and ISRE within introns compared to SNVs predicted to result in deleterious non-synonymous amino acid changes (Fig. 2b). This is consistent with previous observation that genetic variants occur more frequently in the miRNA target regions, compared to the functional regions within miRNAs [62]. In addition, Hsiao et al. demonstrated that alternative splicing events regulated by intronic genetic variants tend to be under positive selection [63], which is consistent with our results that intronic SNVs that potentially affect splicing mechanisms show enrichment of RPS SNVs, compared to the other functional classes (Fig. 2d). On the other hand, splicing factors are more conserved during evolution [63], and our study demonstrates that UCEs were enriched in the genes involved in RNA splicing (Fig. 3b).

On the other hand, genes that modulate response to environmental changes are the most polymorphic. The immune response *MHC* class I and class II genes, implicated in the pathogenesis of several autoimmune diseases, reside in the most polymorphic region of the human genome [36, 64] and carry the highest density of SNVs (Fig. 3c). Notably, this family of genes is significantly enriched in SNVs predicted to alter various regulatory elements including TFBS, ESE/ESS, ISRE and miRBS rather than protein function (Fig. 3e). In fact, while none of the RPS pfSNVs in the *MHC* family is predicted to cause a deleterious effect on protein function, 87 pfSNVs are found to display signature of RPS (Additional file 1: Table S8). Hence, the regulatory regions of the *MHC* family of genes are likely to be under strong positive selection, as previously suggested [65], and are functionally significant, regulating gene expression to modulate phenotypes. For example, a very well-studied polymorphism, rs9378249 upstream of the *HLA-B* gene, has previously been associated with bipolar disorder [66, 67] and hypertension [67]. This polymorphism is predicted to alter TFBS and exhibits the signature of RPS; hence, it may be a causal variant for the various diseases although the underlying molecular mechanism requires further validation.

Another class of genes that modulates response to the environment is the drug/xenobiotic response families of genes including the *ABC* transporter and the *CYP450* metabolism families of genes. Unlike the *MHC* immune genes, which are significantly enriched in regulatory SNVs predicted to modulate gene expression, these drug response gene families are enriched in SNVs that affect the functions of the proteins, namely nsSNVs predicted to be deleterious (Fig. 4b). Previous reports also highlighted the high SNV density and excess of rare nsSNVs of the *CYP450* pathway [68, 69] with 90–95% individuals carrying at least one actionable variant in *CYP450* genes [70]. Another report predicted that ~ 32% (1949/6165) of SNVs at the *CYP450* loci are putatively functional with *CYP4F12* carrying amongst the most novel putatively functional variants [71] which is consistent with our observations that *CYP4F12* is enriched with the highest number of pfSNVs (Additional file 1: Table S9). In addition to the pfSNVs, RPS and highly population differentiated (*F*_ST_ > 0.3) SNVs are significantly represented in the *ABC* transporter genes but not in the *CYP450* genes (Fig. 4d) suggesting that the *ABC* transporter genes may be under stronger positive selection than the *CYP450* genes. For example, rs17822931, a coding variant at the *ABCC11* earwax determinant gene [59], is found to be highly differentiated amongst populations, and the *A* allele is positively associated with adaptation to cold climate [72]. Greater than 40% of these RPS and/or population differentiated SNVs in the *ABC* transporter genes have been associated with phenotype modulation and even diseases, e.g. Alzheimer’s and Schizophrenia (table in Fig. 4e). Nearly all the coding SNVs at the *ABC* transporter gene family that display the signature of RPS or are significantly population differentiated are also predicted to alter ESE/ESS suggesting that differential splicing may also play an important role in the *ABC* genes to generate diverse splicing forms to respond to different environment.

Hence, adaptive genes that respond to environmental changes are likely to be highly polymorphic and subjected to strong positive selection pressures consistent with previous reports that variants associated with inflammatory diseases show evidence of RPS [73], and genes associated with pharmacogenomics show higher level of population differentiation, as a signature of positive selection [74]. Regulation of gene expression through variants that alter TFBS in the *MHC* gene family as well as modulation of protein function and/or splicing pattern in the *ABC* gene families highlight the different ways by different families of genes to adapt to the environment.

## Conclusions

In conclusion, this study elucidates the overall architecture of the genetic polymorphisms, namely SNVs and INDELs, in the human genome. The coding region is found to be under strong negative selection, as being the least population differentiated, showing lowest densities of SNVs and INDELs (especially frame-shift INDELs), the highest proportion of rare alleles with less enrichment of RPS SNVs. SNVs predicted to be functional are found to be under negative selection with enrichment of rare alleles. Families of genes which are ‘master regulators’ of gene expression including those involved in epigenetics, transcription, translation or splicing are found to be least polymorphic, functionally conserved and/or enriched with ultra-conserved elements. Finally, genes that modulate response to the environment are the most polymorphic with the *MHC* gene family, which is involved in immune response, being the most polymorphic while genes involved in drug/xenobiotic response, including *ABC* transporter and *CYP450* genes, are the most enriched with functional nsSNVs.

## Methods

### Polymorphisms in the human genome

Polymorphisms from the dbSNP database (Build 131) were mapped to different genic regions (5′UTRs, coding regions, introns and 3′UTRs) of the human genes (NCBI Genome Build 37.1) with those residing outside genes classified as intergenic variants.

To minimize false-positive SNPs originating from highly paralogous sequences, which were estimated to be ~ 8% of biallelic coding SNVs in dbSNP129 [75], only polymorphisms, which mapped to a single location in the genome and have been validated using a non-computational method or have allele frequency information (e.g. from 1000 Genomes project), were included in this study. In the 1000 genomes project, the variant assignment was restricted to ‘accessible genome’, whereby ambiguously placed reads or unexpectedly high or low numbers of aligned reads were excluded (~ 15% genome) to minimize the detection of false-positive variants [76]. To evaluate if our data is valid, SNV density data of this study was compared and found to be comparable to the SNV density data calculated from whole-genome sequencing of 179 HapMap individuals [76] of 1000 genomes project. For example, similar to our observations using dbSNP data, the MHC gene loci from the 1000 genomes sequencing data were also found to be significantly more polymorphic than other human genes (*p* < 0.001 by Mann-Whitney test). Hence, results from sequencing data from the 1000 genomes project were consistent with the findings in this study using dbSNP data, suggesting that, in spite of the potential ascertainment biases and sequencing artefacts inherent in the dbSNP database, our findings about the enrichment of SNPs in MHC genes are valid.

Two major forms of genetic polymorphisms, SNVs and INDELs, were investigated. SNV/INDEL density within a particular genic region, e.g. 5′UTR, was calculated as the number of SNVs/INDELs divided by the length of that region. For genes with multiple transcripts, the mean densities were taken. Genes lacking polymorphism in all genic regions (promoter, 5′UTR, coding, intron, 3′UTR) were regarded as non-polymorphic genes. Highly polymorphic genes were identified based on a binomial model as described in Additional file 1.

Allele frequency of SNVs in the human genome was determined in the three population (East Asian, African and European) groups (HapMap release 28) as described in Additional file 1.

*F*_ST_statistics [28] using the pooled allele frequencies in the three population groups was then calculated for each of the genotyped and polymorphic loci. Two groups of SNVs, namely, (1) zero-*F*_ST_ SNVs (*F*_ST_ = 0) and (2) high-*F*_ST_ SNVs (*F*_ST_ > 0.3), were further analysed. The fold enrichment of zero-*F*_ST_ or high-*F*_ST_ SNVs in a specific genic region (e.g. coding region) was determined by calculating the percentage of these SNVs in the coding region divided by the percentage of all the genotyped SNVs in the same region, and the significance of enrichment is determined using the Fisher’s exact test. Fold enrichment, which significantly deviates from one, indicates that these SNVs are under- or over-represented in these regions.

### Natural selections

Genic regions that display signatures of negative selection were previously reported to have excess rare derived alleles [31]. Hence, to identify the regions of genes subjected to negative selection, we determined if there is a statistical enrichment of rare SNVs (DAF < 0.05) in each genic region using the Fisher’s exact test.

SNVs displaying signatures of RPS were identified using LD- or haplotype-based methods as described in [26, 77]. To identify the regions enriched in RPS SNVs, the percentage of RPS SNVs within the region was compared with the percentage of RPS SNVs in the whole genome, and significance of difference was determined using the Fisher’s exact test.

UCEs are sequences within the genome that are 100% identical to the sequences with the mouse and the rat genomes [35], hence displaying evolutionary conservation and signatures of strong negative selection. A total of 481 UCEs have been identified [35], of which 70 are evolutionarily conserved coding sequences, overlapping with coding regions.

### Potential functions of SNVs

The pfSNP database (http://pfs.nus.edu.sg/) [26], which integrates a variety of bioinformatics prediction algorithms, was used to evaluate potential functions of all the SNVs in the human genome that alter TFBS, protein functions, splicing events and miRBS. The prediction algorithms employed in this study are described in [26] and Additional file 1.

### Functional annotation

The Database for Annotation, Visualization and Integrated Discovery [78, 79] was utilized for functional annotation of the genes of interest. The enrichment of the genes in PANTHER protein family, GO-molecular function, GO-biological process and KEGG pathway was investigated. Benjamini-Hochberg-corrected *p* value < 0.05 signifies statistical significance.

## Additional file

Additional file 1: Supplementary Materials and Methods as well as **Tables S1-S9**. (PDF 830 kb)

## Abbreviations

3′UTR: 3′ Untranslated region
5′UTR: 5′ Untranslated region
*ABC*: ATP-binding cassette
*CYP450*: Cytochrome P450
DAF: Derived allele frequency
ESE/ESS: Exon splicing enhancer or silencer
*HBB*: Beta haemoglobin
INDEL: Insertion/deletion
ISRE: Intronic splicing regulatory element
*MHC*: Major histocompatibility complex
miRBS: MicroRNA binding site
miRNA: MicroRNA
nfSNV: Non-functional SNV
NMD: Nonsense-mediated decay
nsSNV: Non-synonymous SNV
*OR*: Olfactory receptor
pfSNV: Potentially functional SNV
RPS: Recent positive selection
SNV: Single-nucleotide variant
sSNV: Synonymous SNV
TFBS: Transcription factor binding site
UCE: Ultra-conserved element
