# Supplementary material for: Architecture of polymorphisms in the human genome reveals functionally important and positively selected variants in immune response and drug transporter genes
Source: Hum Genomics. 2018 Sep 15;12:43. doi: 10.1186/s40246-018-0175-1 (PMC6139121; doi:10.1186/s40246-018-0175-1)
Supplement: Supplementary file 1 — Supplementary Materials and Methods as well as Tables S1-S9. (PDF 830 kb) [file 40246_2018_175_MOESM1_ESM.pdf]

Supplemental Information

Architecture of Polymorphisms in the Human Genome reveals Functionally Important and Positively Selected Variants in Immune-Response and Drug Transporter Genes

Yu Jin[1,3], Jingbo Wang[2], Maulana Bachtiar[2,3], Samuel S. Chong[4] and Caroline G. L. Lee [1,2,3,5,*]

*Corresponding E-Mail: bchleec@nus.edu.sg

This file includes:

Supplementary Materials and Methods	Page 1-3
Table S1.	Page 4
Table S2	Page 5
Table S3	Page 6
Table S4	Page 7
Table S5	Page 8
Table S6	Page 9
Table S7	Page 10
Table S8	Page 11
Table S9	Page 12

Supplementary Materials and Methods

Identification of highly polymorphic SNVs

Highly polymorphic genes were defined as those genes which meet the following two criteria: 1) their SNV densities are amongst the top 5% of all genes in the human genome; 2) the observed number of polymorphisms in the entire gene length is statistically significantly higher than the expected number based on a binomial model after Bonferroni correction. The density of SNVs in the human genome was determined to be 4.43 SNVs/kb (total number of 13,727,044 SNVs/total length of 3,100,875,916 bps genome). Based on this density, the expected number of SNVs for a specified length of the gene-of-interest could be determined and compared with the observed number of SNVs in that gene. Only genes with corrected p-values of less than 0.05 by binomial test are considered to have satisfied the second criterion.

Allele frequencies and population differentiation

Allele frequency data were downloaded from HapMap release 28. Nine of the eleven global populations were combined into three population groups according to their geographical origins: East Asian group which includes CHB (Han Chinese in Beijing, China), CHD (Chinese in Metropolitan Denver, Colorado) and JPT (Japanese in Tokyo, Japan), European group which comprises CEU (Utah residents with ancestry from northern and western Europe) and TSI (Toscani in Italia) and the African group which comprises LWK (Luhya in Webuye, Kenya), ASW (African ancestry in Southwest USA), MKK (Maasai in Kinyawa, Kenya) and YRI (Yoruba in Ibadan, Nigeria). For each bi-allelic SNVs, the ancestral allele information was retrieved from the SNPAncestralAllele table in dbSNP database (Build 131). and defined as the allele identical to the chimpanzee allele in the aligned sequence, while the other human allele was named as the derived allele, whose frequencies in three population groups were calculated.

Prediction of potential functions of SNVs

Promoter SNVs that potentially affect transcription factor binding sites

SNVs in putative promoters, which were defined as 5 kb upstream transcription start sites were assessed for their potential functions to alter putative TFBS. Using the tool MATCHTM in TRANSFAC database [1-2], the sequences containing the promoter SNVs were scanned for potential TFBS, as obtained from TRANSFAC 11.3 database. SNVs with alternative alleles that correspond to different binding sites were identified to be potentially functional.

Coding SNVs that potentially affect protein function and/or splicing events

NsSNVs that are potentially deleterious to protein function were obtained from three sources: Polyphen-2 database (http://genetics.bwh.harvard.edu/pph2/) [3], LS-SNP database (http://modbase.compbio.ucsf.edu/LS-SNP/) [4] and SNPs3D database (http://www.snps3d.org/) [5].

Coding SNVs were also evaluated for their potential to cause NMD. Nonsense SNVs coding for a premature stop codon in any exon except the last one and is more than 50 bps away from the intron-exon boundary were predicted to cause NMD [6].

For identification of SNVs that either create or destroy potential ESE or ESS sites, an algorithm developed in PS Scan (ftp://ftp.expasy.org/databases/prosite/tools/ps_scan) which is capable of identifying motifs from sequences in batch format was adopted. All the possible ESE and ESS motifs were downloaded from the supplementary information of various publications [7-9], converted to Prosite compatible format and then uploaded onto the PS Scan program together with information with regards to the sequences around the coding SNVs. The PS scan program

then scanned these sequences for possible ESE/ESS motifs. SNVs having alternative alleles with different putative ESE/ESS patterns were determined to be potentially functional.

Intronic SNVs that potentially affect splicing events

Intronic SNVs within 400 bps of exon-intron boundaries were assessed for their potential functions to create or destroy ISREs using PS scan program and possible ISRE motifs described in a previous study [10]. SNVs that alter the consensus sequence of putative ISREs were classified as pfSNVs.

3'UTR SNVs that potentially affect miRNA binding sites

SNVs predicted to alter miRNA targets were retrieved from PolymiRTS (http://compbio.uthsc.edu/miRSNP/) [11] (Bao et al., 2007) and Patrocles (http://www.patrocles.org/Patrocles_targets.htm) [7] databases.

1. Matys V, Kel-Margoulis OV, Fricke E, Liebich I, Land S, Barre-Dirrie A, et al. TRANSFAC and its module TRANSCompel: transcriptional gene regulation in eukaryotes. *Nucleic Acids Res*. 2006;**34**:D108-10.
2. Matys V, Fricke E, Geffers R, Gossling E, Haubrock M, Hehl R, et al. TRANSFAC: transcriptional regulation, from patterns to profiles. *Nucleic Acids Res*. 2003;**31**:374-8.
3. Adzhubei IA, Schmidt S, Peshkin L, Ramensky VE, Gerasimova A, Bork P, et al. A method and server for predicting damaging missense mutations. *Nat Methods*. 2010;**7**:248-9.
4. Karchin R, Diekhans M, Kelly L, Thomas DJ, Pieper U, Eswar N, et al. LS-SNP: large-scale annotation of coding non-synonymous SNPs based on multiple information sources. *Bioinformatics*. 2005;**21**:2814-20.
5. Yue P, Melamud E, Moult J. SNPs3D: candidate gene and SNP selection for association studies. *BMC Bioinformatics*. 2006;**7**:166.
6. Nagy E, Maquat LE. A rule for termination-codon position within intron-containing genes: when nonsense affects RNA abundance. *Trends Biochem Sci*. 1998;**23**:198-9.
7. Fairbrother WG, Yeh RF, Sharp PA, Burge CB. Predictive identification of exonic splicing enhancers in human genes. *Science*. 2002;**297**:1007-13.
8. Wang Z, Rolish ME, Yeo G, Tung V, Mawson M, Burge CB. Systematic identification and analysis of exonic splicing silencers. *Cell*. 2004;**119**:831-45.
9. Zheng ZM. Regulation of alternative RNA splicing by exon definition and exon sequences in viral and mammalian gene expression. *J Biomed Sci*. 2004;**11**:278-94.
10. Yeo GW, Van Nostrand EL, Liang TY. Discovery and analysis of evolutionarily conserved intronic splicing regulatory elements. *PLoS Genet*. 2007;**3**:e85.
11. Bao L, Zhou M, Wu L, Lu L, Goldowitz D, Williams RW, et al. PolymiRTS Database: linking polymorphisms in microRNA target sites with complex traits. *Nucleic Acids Res*. 2007;**35**:D51-4.

Table S1. Functional annotation of non-polymorphic genes.

Category	Term	# genes	Fold Enrichment	Correcated P-value
Protein family	Histone H2B	5	107.27	3.82E-06
	Histone H2A	5	90.33	4.04E-06
Function	DNA binding	18	2.94	8.14E-04
Process	Nucleosome assembly	11	53.92	5.51E-13
Pathway	Systemic lupus erythematosus	11	29.64	1.96E-12

Table S2. Functional annotation of genes without nsSNVs or INDELs in their coding regions.

Category	Term	# genes	Fold Enrichment	Corrected P value
Protein Family	Ras-related GTPase	57	2.86	3.20E-11
	Homeobox protein	57	2.43	5.08E-08
	Histone H2A	13	4.4	1.52E-03
	Cis-golgi matrix protein GM130	9	5.26	1.21E-02
	Similar to preferentially expressed antigen in melanoma-like 3	12	3.68	2.17E-02
	Guanine nucleotide-binding protein gamma subunit	9	4.83	2.49E-02
	Histone H2B	10	4.02	4.01E-02
	Ubiquitin-conjugating enzyme E2	16	2.71	4.63E-02
Function	Transcription factor activity	222	1.55	1.38E-09
	GTPase activity	64	2.06	2.81E-06
	Structural constituent of ribosome	48	2.07	1.06E-04
	Cytokine activity	56	1.92	1.51E-04
	Hydrogen ion transmembrane transporter activity	24	1.93	1.64E-01
Process	RNA splicing	96	2.2	5.57E-11
	Nucleosome assembly	35	2.95	1.31E-06
	Translational elongation	41	2.66	1.46E-06
	Small GTPase mediated signal transduction	82	1.76	5.51E-05
	Regulation of transcription	476	1.21	1.99E-04
	Pattern specification process	66	1.6	1.22E-02
	Neuron fate commitment	17	2.63	3.89E-02
Pathway	Spliceosome	52	2.8	1.46E-10
	Systemic lupus erythematosus	35	2.4	3.96E-05
	Chemokine signaling pathway	50	1.81	9.24E-04
	Oxidative phosphorylation	35	1.98	2.60E-03
	Cardiac muscle contraction	24	2.2	7.18E-03
	Cytokine-cytokine receptor interaction	58	1.5	2.67E-02

Table S3. Functional annotation of genes containing ultra-conserved elements in their coding regions.

Category	Term	# genes	Fold Enrichment	Correcated P value
Protein Family	Homeobox protein	9	14.88	7.37E-06
Function	Transcription factor activity	22	4.93	3.36E-08
Process	Regulation of transcription	32	2.85	8.18E-07
	RNA splicing	12	9.66	4.25E-06
	Embryonic morphogenesis	12	8.9	5.59E-06
	Pattern specification process	8	6.82	3.35E-03
Pathway	Spliceosome	5	13.41	5.99E-03

Table S4. Functional annotation of highly polymorphic genes.

Category	Term	# genes	Fold Enrichment	Corrected P value
Protein Family	MHC Class II-related	11	2.17	8.15E-12
	Cytochrome P450	13	2.57	6.14E-07
	MHC Class I-related	6	1.19	1.19E-02
	Interleukin-1-related	4	0.79	2.92E-02
	C-type lectin superfamily member	5	0.99	3.04E-02
Function	MHC class II receptor activity	10	1.98	3.66E-08
	MHC class I receptor activity	7	1.38	1.03E-04
	Aromatase activity	8	1.58	1.09E-04
	Electron carrier activity	17	3.36	2.74E-03
	Interleukin-1 receptor antagonist activity	4	0.79	1.27E-02
Process	Immune response	49	9.68	3.26E-09
	Oxidation reduction	36	7.11	9.09E-04
	Blood circulation	17	3.36	3.46E-03
	Defense response	31	6.13	3.62E-02
Pathways	Graft-versus-host disease	19	3.75	3.34E-16
	Antigen processing and presentation	22	4.35	6.05E-13
	Type I diabetes mellitus	17	3.36	4.34E-13
	Allograft rejection	16	3.16	4.33E-13
	Autoimmune thyroid disease	17	3.36	9.90E-12
	Viral myocarditis	17	3.36	1.97E-09
	Asthma	11	2.17	9.95E-08
	Cell adhesion molecules	17	3.36	1.94E-05
	Systemic lupus erythematosus	13	2.57	4.31E-04
	Drug metabolism	10	1.98	9.62E-04
	Arachidonic acid metabolism	9	1.78	2.50E-03
	Caffeine metabolism	4	0.79	8.16E-03
	Natural killer cell mediated cytotoxicity	12	2.37	1.72E-02
	Steroid hormone biosynthesis	7	1.38	1.90E-02
	Hematopoietic cell lineage	9	1.78	3.06E-02

Table S5. Functional annotation of genes affected by >5 deleterious nsSNVs

Category	Term	# genes	Fold Enrichment	Corrected P value
Protein family	ATP-binding cassette transporter	14	9.88	6.32E-08
	Tyrosine protein kinase	24	4.23	1.53E-06
	Dynein heavy chain	10	11.59	3.60E-06
	Spectrin-like cell structure protein	16	5.53	1.06E-05
	Myosin	15	5.41	3.30E-05
	Laminin	11	5.95	6.02E-04
	Intermediate filament	17	3.63	8.99E-04
	EGF-like domain protein	15	3.53	4.24E-03
	Low density lipoprotein receptor	8	6.18	1.03E-02
	Cytochrome p450	12	3.42	3.21E-02
Pathway	ABC transporters	23	7.5	1.47E-12
	ECM-receptor interaction	23	3.93	2.48E-06
	Focal adhesion	36	2.58	1.16E-05
	Viral myocarditis	18	3.75	1.22E-04
	Complement and coagulation cascades	15	3.12	7.15E-03
	Mismatch repair	8	4.99	1.60E-02
	Caffeine metabolism	5	10.25	1.86E-02

Table S6. Functional annotation of genes with SNVs that cause nonsense-mediated decay.

Category	Term	# genes	Fold Enrichment	Corrected P-value
Function	ATPase activity	35	2.51	1.17E-04
	Primary active transmembrane transporter activity	15	2.97	2.78E-02
Process	M phase	35	2.59	1.80E-03
	DNA metabolic process	42	2.07	9.09E-03
	Cell cycle	57	1.8	1.14E-02
	Sexual reproduction	38	2.04	2.35E-02
Pathway	ABC transporters	12	6.04	3.88E-04

Table S7. Functional analysis of genes with coding INDELs that cause frame-shift.

Category	Term	# genes	Fold Enrichment	Corrected P-value
Protein Family	Cytochrome P450	8	14.45	1.66E-04
Function	Oxidoreductase activity, acting on paired donors, with incorporation or reduction of molecular oxygen, reduced flavin or flavoprotein as one donor, and incorporation of one atom of oxygen	8	28.43	3.17E-06
	Aromatase activity	7	27.86	2.54E-05
	Electron carrier activity	10	4.74	1.37E-02
Process	Oxidation reduction	19	3.1	4.56E-02
Pathway	Metabolism of xenobiotics by cytochrome P450	8	12.29	2.41E-04
	Drug metabolism	7	10.4	1.97E-03

Table S8. The MHC class I and class II genes investigated in this study.

MHC class	Gene Symbol	Gene Name	SNV density					# SNVs			
			Genic	5'UTR	coding	intron	3'UTR	Total	pfSNVs	*RPS pfSNVs	RPS deleterious nsSNVs
I	HFE	Hemochromatosis	6.05	6.25	5.88	6.21	4.38	48	24	3	0
	HLA-A	major histocompatibility complex, class I, A	62.27		84.70	54.88	35.55	222	177	0	0
	HLA-B	major histocompatibility complex, class I, B	55.97	18.52	78.05	43.53	55.94	197	148	2	0
	HLA-C	major histocompatibility complex, class I, C	62.22	142.86	77.20	53.27	59.95	245	167	17	0
	HLA-E	major histocompatibility complex, class I, E	5.75	26.32	7.43	4.21	6.96	22	27	1	0
	HLA-F	major histocompatibility complex, class I, F	10.36	8.06	6.61	12.88	0.00	46	41	3	0
	HLA-G	major histocompatibility complex, class I, G	17.86	5.62	14.75	19.10	23.50	75	54	0	0
	MICA	MHC class I polypeptide-related sequence A	20.31	25.64	46.01	17.09	40.23	244	87	8	0
	MICB	MHC class I polypeptide-related sequence B	12.11	25.86	7.81	13.35	4.12	165	46	3	0
II	HLA-DMA	major histocompatibility complex, class II, DM alpha	5.99	0.00	11.45	4.72	8.00	27	14	3	0
	HLA-DMB	major histocompatibility complex, class II, DM beta	9.37	8.58	10.10	8.92	15.02	60	21	0	0
	HLA-DOA	major histocompatibility complex, class II, DO alpha	11.97	13.16	21.25	12.33	9.04	65	42	10	0
	HLA-DOB	major histocompatibility complex, class II, DO beta	11.20	20.62	10.95	10.98	11.04	48	44	13	0
	HLA-DPA1	major histocompatibility complex, class II, DP alpha 1	34.36	0.00	38.31	33.52	46.65	298	58	6	0
	HLA-DPB1	major histocompatibility complex, class II, DP beta 1	29.60	0.00	48.91	26.97	48.12	379	113	12	0
	HLA-DQA1	major histocompatibility complex, class II, DQ alpha 1	93.32	94.34	93.75	85.02	147.02	622	319	0	0
	HLA-DQA2	major histocompatibility complex, class II, DQ alpha 2	24.76	51.72	27.34	25.06	19.25	154	80	3	0
	HLA-DQB1	major histocompatibility complex, class II, DQ beta 1	91.63	73.17	129.77	86.65	89.89	648	379	0	0
	HLA-DRA	major histocompatibility complex, class II, DR alpha	15.84	24.69	6.54	16.82	22.17	82	46	5	0
	HLA-DRB1	major histocompatibility complex, class II, DR beta 1	75.88	186.05	113.61	70.55	130.43	852	343	0	0
	HLA-DRB3	major histocompatibility complex, class II, DR beta 3	0.84	0.00	3.75	0.50	6.31	11	3	0	0
	HLA-DRB4	major histocompatibility complex, class II, DR beta 4	3.67	0.00	4.99	2.83	37.38	57	0	0	0
	HLA-DRB5	major histocompatibility complex, class II, DR beta 5	10.42	0.00	21.22	9.67	10.96	135	19	0	0

* In total, 87 RPS pfSNVs were identified in the MHC genes, including rs2071353 and rs2071351 that alter both TFBS in HLA-DPA1 and ISRE in HLA-DPB1.

Table S9. Summary of pfSNVs in CYP450 genes.

Gene	# pfSNVs
CYP4F12	71
CYP1B1	67
CYP4V2	64
CYP3A4	57
CYP2A7	55
CYP2C9	54
PTGIS	49
CYP1A2	49
CYP1A1	47
CYP11B2	47
CYP2B6	47
CYP19A1	45
CYP4B1	45
CYP4F3	44
CYP2C8	44
CYP24A1	43
CYP2E1	42
CYP11B1	41
CYP2D6	40
CYP2A6	40
CYP2C19	40
CYP4A11	39
CYP3A5	39
TBXAS1	39
CYP4F8	37
CYP3A7	33
CYP2J2	33
CYP17A1	31
CYP3A43	31
CYP4F11	29
CYP4Z1	27
CYP4A22	26
CYP21A2	26
CYP39A1	25
CYP4X1	24
CYP2S1	21
CYP4F2	21
CYP2C18	21
CYP2F1	20
CYP2A13	20
CYP8B1	20
CYP46A1	19
CYP11A1	18
CYP7A1	17
CYP20A1	16
CYP4F22	15
CYP26C1	14
CYP2U1	14
CYP27C1	13
CYP27A1	12
CYP27B1	11
CYP51A1	11
CYP26A1	11
CYP26B1	10
CYP2R1	7
CYP2W1	7
CYP7B1	5
